# THE RELATIONSHIP BETWEEN EVACUATION AND HEALTH CARE OUTCOMES AMONG ASSISTED LIVING RESIDENTS AFTER HURRICANE IRMA

**DOI:** 10.1093/geroni/igae098.0755

**Published:** 2024-12-31

**Authors:** Cassandra Hua, Sweta Patel, Kali Thomas, Dylan Jester, Lindsay Peterson, Debra Dobbs, Ross Andel, David Dosa

**Affiliations:** University of Massachusetts Lowell, Lowell, Massachusetts, United States; Brown University, Providence, Rhode Island, United States; Johns Hopkins University, Baltimore, Maryland, United States; VA Palo Alto Healthcare System, Palo Alto, California, United States; University of South Florida, Tampa, Florida, United States; University of South Florida, Tampa, Florida, United States; Arizona State University, Phoenix, Arizona, United States; Brown University, Providence, Rhode Island, United States

## Abstract

Evacuation is associated with adverse outcomes among nursing home residents during hurricanes, but the impact on assisted living (AL) residents remains unknown. This retrospective cohort study examined the relationship between evacuation and healthcare outcomes (i.e., emergency department visits, hospitalizations, mortality, and nursing home visits) among Florida AL residents exposed to Hurricane Irma. We identified a cohort of Florida AL residents who were 65+ years old, enrolled in Medicare Fee-For-Service, and resided in 9-digit ZIP codes corresponding to US assisted living communities with ≥25 beds on September 10th, 2017, the day of Hurricane Irma’s landfall. We performed 1:4 propensity score matching. Using the matched sample, we examined the relationship between evacuation status and health outcomes within multilevel logistic regression models with a random intercept. Models adjusted for neighborhood disadvantage and distance to the storm trajectory. Of 25,130 Florida AL residents in our cohort, 3,402 (13.5%) evacuated and 21,728 (86.5%) did not evacuate. When compared to sheltering-in-place, evacuation was associated with a 16% greater odds of emergency department visits (adjusted odds ratio [AOR]=1.16, p=0.037, 95% CI 1.01-1.33) and 51% greater odds of nursing home visits (AOR 1.51, 95% CI: 1.14-2.00) within 30 days of Hurricane Irma’s landfall. Hospitalization and mortality did not vary significantly by evacuation status within 30 or 90 days after the landfall date. Stress caused by evacuation may yield poorer immediate health outcomes after a major storm for AL residents. Potential benefits and harms of evacuating vs sheltering-in-place must be carefully considered when developing emergency planning and response.

